# HSP70 Enhances Immunosuppressive Function of CD4^+^CD25^+^FoxP3^+^ T Regulatory Cells and Cytotoxicity in CD4^+^CD25^−^ T Cells

**DOI:** 10.1371/journal.pone.0051747

**Published:** 2012-12-26

**Authors:** Julian Wachstein, Sabine Tischer, Constanca Figueiredo, Anne Limbourg, Christine Falk, Stephan Immenschuh, Rainer Blasczyk, Britta Eiz-Vesper

**Affiliations:** 1 Institute for Transfusion Medicine, Hannover Medical School, Hannover, Germany; 2 Integrated Research and Treatment Center (IFB-Tx), Hannover Medical School, Hannover, Germany; 3 Department of Plastic, Hand and Reconstructive Surgery, Hannover Medical School, Hannover, Germany; 4 Institute for Transplant Immunology, Hannover Medical School, Hannover, Germany; National Cancer Institute (INCA), Brazil

## Abstract

Human CD4^+^CD25^+^FoxP3^+^ T regulatory cells (Tregs) control effector T cells and play a central role in peripheral tolerance and immune homeostasis. Heat shock protein 70 (HSP70) is a major immunomodulatory molecule, but its effect on the functions of Tregs is not well understood. To investigate target-dependent and –independent Treg functions, we studied cytokine expression, regulation of proliferation and cytotoxicity after exposure of Tregs to HSP70. HSP70-treated Tregs significantly inhibited proliferation of CD4^+^CD25^−^ target cells and downregulated the secretion of the proinflammatory cytokines IFN-γ and TNF-α. By contrast, HSP70 increased the secretion of Treg suppressor cytokines IL-10 and TGF-β. Treatment with HSP70 enhanced the cytotoxic properties of Tregs only to a minor extent (4-fold), but led to stronger responses in CD4^+^CD25^−^ cells (42-fold). HSP70-induced modulation of T-cell responses was further enhanced by combined treatment with HSP70 plus IL-2. Treatment of Tregs with HSP70 led to phosphorylation of PI3K/AKT and the MAPKs JNK and p38, but not that of ERK1/2. Exposure of Tregs to specific inhibitors of PI3K/AKT and the MAPKs JNK and p38 reduced the immunosuppressive function of HSP70-treated Tregs as indicated by the modified secretion of specific target cell (IFN-γ, TNF-α) and suppressor cytokines (IL-10, TGF-β). Taken together, the data show that HSP70 enhances the suppressive capacity of Tregs to neutralize target immune cells. Thus HSP70-enhanced suppression of Tregs may prevent exaggerated immune responses and may play a major role in maintaining immune homeostasis.

## Introduction

Heat shock proteins (HSPs) are a highly conserved group of cytoprotective proteins which are typically up-regulated in response to various stress stimuli. HSPs have been identified in a wide variety of prokaryotic and eukaryotic cells. They play an important role as intracellular molecular chaperones that prevent the aggregation and folding of proteins [1], [2]. HSPs can further act at multiple points in apoptotic pathways to ensure that stress-induced damage does not inappropriately trigger cell death [3], [4]. Knowledge of their immunological functions emerged with the observation that HSP70, HSP90, gp96, calreticulin, HSP110 and GRP170 isolated from tumor cells can initiate adoptive, tumor-specific T-cell responses and protective immunity, whereas those from healthy cells do not [5], [6]. Human heat shock protein 70 (HSP70) has potent immunomodulatory properties and has also been shown to regulate the activity of T cells [7].

Naturally occurring CD4^+^CD25^+^ T regulatory cells (Tregs) play a major role in immunoregulatory responses. Although these cells are known to suppress T-cell proliferation *in vitro*, they may also inhibit immune responses to autoantigens, alloantigens, tumor antigens and infectious agents *in vivo* [8], [9]. Tregs have been found to control effector T cells in animal models of autoimmune diseases, while a decrease in the number and function of Tregs has been reported in humans with autoimmune diseases such as type 1 diabetes and multiple sclerosis [10]–[14].

Tregs express a variety of specific surface markers, such as CD25 (IL-2Rα-chain), CD62L, CTLA-4 (CD152), and glucocorticoid-induced tumor necrosis factor receptor family-related protein (GITR) [15]–[17]. The transcription factor forkhead box P3 (FoxP3) plays a key role in the development and function of Tregs [18], [19]. Mutations in the gene encoding FoxP3 lead to immune dysregulation and polyendocrinopathy, enteropathy X-linked (IPEX) syndrome, a disorder characterized by severe multiple autoimmune diseases [20].

Little is known on how HSPs may influence the suppressive function of Tregs. Human HSP60 has been found to downregulate T-cell migration by interacting with TLR2 and to inhibit the secretion of proinflammatory cytokines by activated T cells [21], [22]. Tregs were recently identified as the T-cell subtype responsible for innate HSP60-mediated anti-inflammatory effects [23]. In contrast to HSP60 which is primarily located in the mitochondria, HSP70 is primarily found in the cytosol and in the nucleus. The goal of the present study was to investigate the immunomodulatory effects of extracellular HSP70 on human CD4^+^CD25^+^ T regulatory cells and to evaluate the potential HSP70-mediated cytotoxicity of both, CD4^+^CD25^+^ Tregs and their CD4^+^CD25^−^ target cells.

We demonstrate that HSP70 and in particular the combination of HSP70 plus IL-2 enhances the capacity of Tregs to increase the secretion levels of the immunosuppressive cytokines IL-10, TGF-β, and to decrease the activity CD4^+^CD25^−^ target cells compared to untreated Tregs. We found an increased activity of PI3K/AKT, p38 and JNK signaling pathways upon stimulation with HSP70 in CD4^+^CD25^−^ T cells. Furthermore, we report that the cytotoxic potential of Tregs can be further enhanced by HSP70 as indicated by enhanced granzyme B secretion.

## Materials and Methods

### Expression and purification of recombinant soluble HSP70

In order to facilitate eukaryotic expression and isolation procedures, we previously developed a strategy for expression of soluble HSP70 (HSP70, gene HSPA1A-001) secreted into the cell culture supernatant [24]. The Limulus amebocyte lysate assay (LAL) was used to exclude endotoxin contamination of the purified protein (Rapid Endo-Test, Lonza, Verviers, Belgium, sensitivity 0.005 EU/ml). Endotoxin levels in the HSP70 preparation was determined by LAL assay and were found to be below 0.2 EU/ml (HSP70: 0.15 EU/ml) which has been referred to as endotoxin-free [25], [26]. Recently we were able to demonstrate the T-cell response as specific for recombinant sHSP70 by performing control experiments with proteins which were expressed and isolated under the same conditions (recombinant Sema7A, CMVpp65) [24].

### CD4^+^CD25^+^ Treg and CD4^+^CD25^−^ T-cell isolation and stimulation

CD4^+^CD25^+^ Tregs or CD4^+^CD25^−^ target cells were isolated from peripheral blood collected with the informed consent of healthy platelet donors as approved by the ethics committee of Hannover Medical School. 62% of donors were male (mean age 44.1 years, range 28–64 years) and 48% of donors were female with a mean age of 45.6 years (range 29–60 years). Briefly, the blood samples were incubated with RosetteSep human CD4^+^ T-cell enrichment cocktail (Stemcell Technologies, Grenoble, France) at room temperature for 20 min. Cells were then loaded onto lymphocyte separation medium, isolated by density centrifugation and washed twice with MACS Cell Separation Buffer (Miltenyi Biotec, Bergisch-Gladbach, Germany). In a second round of purification, CD4^+^ T cells were separated into CD25^+^ and CD25^−^ populations with magnetically coupled monoclonal antibodies (mAb) against human CD25 (EasySep Cell Selection, Stemcell Technologies). Purity of CD4^+^CD25^+^ and CD4^+^CD25^−^ T cells was verified by flow cytometry using anti-human CD4 mAb (clone RPA-T4), anti-human CD25 mAb (clone BC96), anti-human FoxP3 (clone 259D), anti-human GITR mAb (CD357, clone 621), and anti-human CTLA-4 (CD152, clone L3D10) (all from BioLegend, San Diego, CA). Data acquisition (FACSCantoII, BD Biosciences, San Jose, CA) and analysis were realized using BD FACSDiva V6.1.2 software (BD Biosciences, Heidelberg, Germany). Staining for FoxP3 and CTLA-4 was performed intracellular according to the manufacturer's instructions using the FOXP3 Fix/Perm Buffer Set (BioLegend). At least 50000 events within the live gate were acquired for each analysis. Only preparation containing CD4^+^CD25^+^ T cells with a purity >80% (range 81.1–96.1%, mean 91.4%) expressing >80% FoxP3 (range 82.5–93.9%, mean 89.5%) and CD4^+^CD25^−^ T cells with a purity >92% (range 92.2–98.1%, mean 95.5%) were used in further experiments. More than 60% of CD4^+^CD25^+^FoxP3^+^ cells stained also positive for CTLA-4 (range 61.1–84.1%, mean 72.6%). Isolated CD4^+^CD25^+^FoxP3^+^ cells expressed low levels of GITR (mean 0.3%, data not shown).

Purified CD4^+^CD25^+^ and CD4^+^CD25^−^ T cells were resuspended at a concentration of 10^6^ cells/ml in RPMI1640 culture medium (Lonza, Basel, Switzerland) supplemented with 10% heat-inactivated human AB serum (C.C.pro, Oberdorla, Germany) and incubated with either IL-2 (200 U/ml), HSP70 (10 µg/ml) or both (cytokines from PeproTech, Rocky Hill, USA) for 2 h, washed, transferred to 96-well plates coated with 1 µg/ml anti-CD3 antibody OKT3 (eBioscience, San Diego, USA) in serum-free medium, and incubated at 37°C. In initial experiments the optimal working concentration for HSP70 was determined (Figure S1). For negative control cells cultured in the absence of any stimuli except of 50 U/ml IL-2 to guarantee survival of the Tregs [27]. A combination of phorbol-12-myristat-13-acetate (PMA) and ionomycin (Iono) at concentrations of 50 ng/ml and 500 ng/ml, respectively, were used as positive controls for the cytokine experiments.

Supernatants and cells were analyzed for cytokine expression after 48 h and after 5 days for proliferation.

### Immunofluorescence staining of Tregs with HSP70-FITC

Three mg purified HSP70 were conjugated with FITC (Fluoro tag FITC conjugation Kit, Sigma-Aldrich, Hamburg, Germany). Tregs were isolated by positive selection as described and purity of the resulting CD25^+^ cells was verified by flow cytometry. 5×10^5^ Tregs (purity >95%) were incubated with 10 µg/ml FITC-labeled HSP70 (HSP70-FITC) and 10 µg/ml PE-labeled CD25 in 500 µl medium (37°C, 5%CO_2_) using 4-well chamber slides (Sigma-Aldrich). After 2 h of culture, the cells were washed carefully, resuspended and fixed in 4% PFA (BD Biosciences). Tregs were carefully washed and stained with 10 µg/ml diamidino-2-phenylindole (DAPI, Invitrogen, Karlsruhe, Germany) for 5 min. Stained cells were washed with PBS, mounted on slides in ProLong Gold Antifade Reagent mounting medium (Invitrogen) and placed under constant illumination on the Olympus-IX81 microscope (Olympus, PA, USA) with a DAPI and FITC filter set using a 60× objective. Images were acquired using a CCD camera (Olympus) and analyzed using Olympus cell^IM^ and cell^IR^ image 3.0 software (Olympus).

### Intracellular FoxP3, caspase-3 and Ki-67 staining

Purified CD4^+^CD25^+^ and CD4^+^CD25^−^ T cells were stimulated for 24 h, washed, fixed for 15 min and permeabilized for 30 min using the Human FoxP3 Buffer Set (BD Bioscience). Cells were then stained with 10 µl/test human FoxP3 (clone 259D/C7), 5 µl/test human Ki-67 (clone MOPC 21) or 5 µl/test human active caspase-3 (clone C92-605) antibody, respectively, and expression was determined by flow cytometry analysis.

### T-cell proliferation assay

Freshly isolated CD4^+^CD25^−^ T cells were labeled with CSFE [5- or 6-(N-succinimidyloxicarbonyl)-3′, 6′-O,O′-diacetylfluorescein)] purchased from Molecular Probes (Eugene, OR, USA) at a final concentration of 1 µM. CD25^−^ cells were then co-cultured with 2 h-prestimulated CD25^+^ Tregs. For control purposes CFSE-labeled CD4^+^CD25^−^ T cells were cultured in the absence of Tregs. After 5 days of incubation, T cells were stained with peridinin chlorophyll protein (PerCP)-conjugated anti-CD4 (BD Biosciences) and cell proliferation of CD4^+^CFSE^+^ target T cells was evaluated by flow cytometry. Supernatant were harvested and analyzed for cytokine secretion.

### Detection of activation of PI3K/AKT pathway, JNK, ERK1/2 and p38 pathways

In order to determine activation of the PI3K/AKT pathway and the p38 MAPKs JNK and ERK1/2, 1×10^6^ cells purified CD4^+^CD25^+^ T cells were stimulated for 10 min with IL-2 (200 U/ml), HSP70 (10 µg/ml) and both. The cells were then washed and replated in the same concentration on anti-CD3 mAb- (OKT3, 1 µg/ml) precoated 24-well plates for 5, 10, 20 and 30 min (37°C, 5%CO_2_). Total cell lysates derived from the cell culture samples were prepared according to the manufacturer's instructions (BioRad, Hercules, USA). Phosphorylation of the 4 target proteins (AKT, p38 MAPK, JNK, ERK1/2) in CD4^+^CD25^+^ T-cell subsets was detected using the bead-based BioPlex phosphoprotein detection assay (BioRad). Samples were analysed on a Luminex-200 instrument using Bio-Plex Manager 6.0 software (BioRad). Untreated CD4^+^CD25^+^ T cells were adjusted to 1.00 by the use of the Bio-Plex Manager 6.0 software (BioRad) and used to calculate the ratios.

### Inhibition of intracellular protein kinases

CD4^+^CD25^+^ T cells were treated with an 5 µM intracellular signal transduction inhibitor (Wortmannin, SB 203580, JNK; all from Calbiochem/EMD Bioscience, Darmstadt, Germany) for 15 min before incubation with IL-2 (200 U/ml), HSP70 (10 µg/ml) and both for 2 h. Cells were then co-cultured with CD4^+^CD25^−^ target T cells (E∶T ratio 1∶5) on 96-well plates coated with anti-CD3 antibodies (OKT3, 1 µg/ml) in serum-free medium for 48 h before analyzing the supernatants for IFN-γ, TNF-α and IL-10 secretion by Luminex Technology and for TGF-β secretion by ELISA. For control, non-preactivated CD4^+^CD25^+^ Tregs were co-cultured with CD4^+^CD25^−^ target cells without prior incubation with the respective inhibitors (control). Increase or decrease of cytokine secretion was expressed as fold-increase in response to the results obtained for the respective control.

### Evaluation of granzyme B, IL-10 and TGF-β mRNA levels by real-time RT-PCR

Granzyme B mRNA levels of both CD4^+^CD25^+^ and CD4^+^CD25^−^ T-cell subsets were evaluated as previously described [24]. Briefly, total cellular RNA was isolated from cells (RNeasy Mini Kit, Qiagen, Hilden, Germany) after 2 days of stimulation with HSP70, IL-2 or both, but without target cells (target-independent). Primers were designed to amplify transcripts of granzyme B mRNA (5′-TGC AAC CAA TCC TGC TTC TG-3′ and 5′-CCG ATG ATC TCC CCT GCA T-3′). One-step real-time PCR (RT-PCR Master Mix, Applied Biosystems, Darmstadt, Germany) was performed using a MGB-TaqMan probe for granzyme B (5′-TGG CCT TCC TCC TGC TGC CCA-3′) [28]. Inventoried mixes (Applied Biosystems) were used for quantification of IL-10 and TGF-β mRNA levels. The constitutively expressed GAPDH gene was used as reference standard for normalization of mRNA levels. Unstimulated CD4^+^CD25^+^ and CD4^+^CD25^−^ T-cells, respectively were used as negative control and the relative quantification (RQ) values for these experiments were adjusted to 1.00. RQ values were calculated by the delta-delta CT method.

### Evaluation of TGF-β and granzyme B secretion by ELISA

TGF-β cytokine secretion was detected by ELISA in the supernatants after 48 h of stimulation according to the manufacturer's instructions (R&D Systems, Minneapolis, USA). Target-independent secretion of granzyme B was detected in the supernatant of the cultures on day 3 after stimulation of the CD4^+^ T-cell populations with the different combinations of HSP70 and IL-2. The protein was assayed by ELISA according to the manufacturer's instructions (Bender MedSystems, Vienna, Austria).

### Multiple cytokine detection

A bead-based multiplexed assay (Luminex Cytokine Human 10-Plex Panel, Invitrogen) that quantifies multiple cytokines in single-sample supernatant was used for analysis of cytokine expression patterns of the cultured T cells. Cytokine detection was performed according to the manufacturer's instructions on a Luminex-200 instrument (Invitrogen).

### Statistics

Statistical analyses were performed using nonparametric Mann-Whitney U and two-tailed t-tests run on GRAPHPAD PRISM V5.02 software (GraphPad Software, San Diego, CA). Levels of significance are expressed as p-values (*p<0.05, **p<0.01, ***p<0.001).

## Results

### Uptake of HSP70 by CD4^+^CD25^+^ Tregs and effect of HSP70 on Ki-67 and caspase-3 expression by CD4^+^ T cells

**Figure 1:**
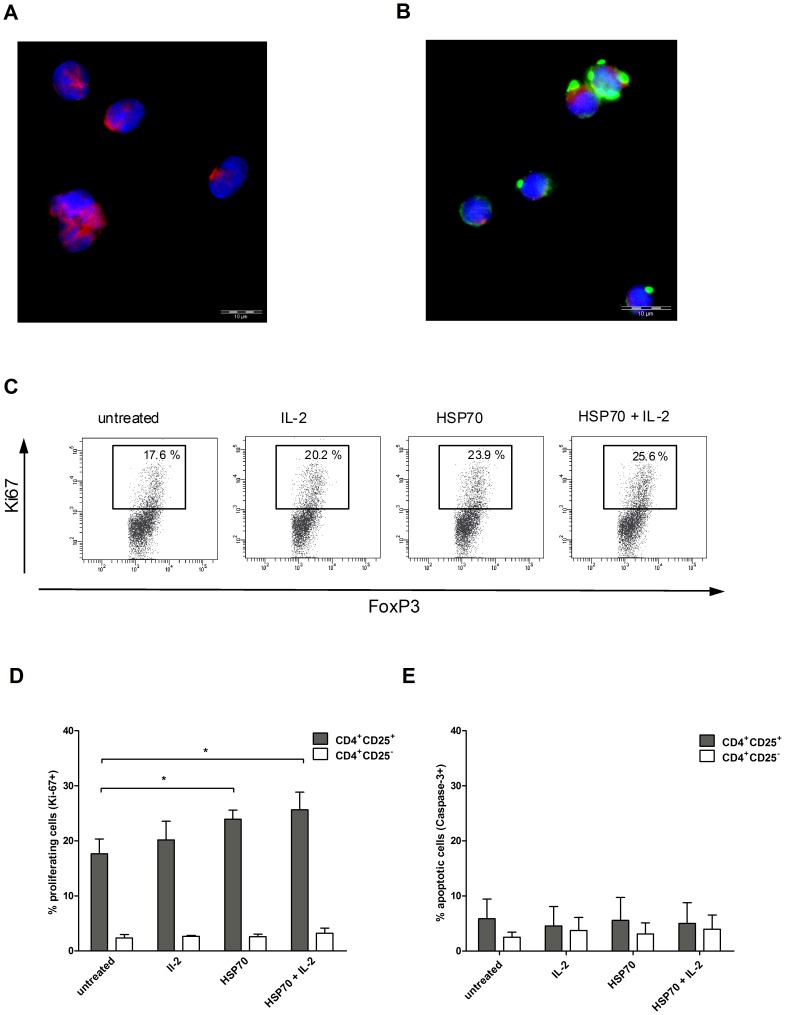
Uptake of HSP70 by CD4^+^CD25^+^ Tregs and HSP70-dependent expression of Ki-67 and caspase-3. Representative fluorescence microscopy results for isolated Tregs incubated for 2 h (**A**) without or with (**B**) HSP70-FITC. Shown are the immunofluorescence microscopy results for DAPI (blue), FITC (green) and PE (red) staining. Purified CD4^+^CD25^+^ and CD4^+^CD25^−^ T cells were incubated with IL-2 (200 U/ml), HSP70 (10 µg/ml) or both for 2 h, washed and transferred to 96-well plates coated with anti-CD3 antibodies (OKT3, 1 µg/ml) in serum-free medium. After 20 h, Ki-67 and caspase-3 expression was determined by FACS analysis. (**C**) Results of one representative Ki-67-experiment out of six in CD4^+^CD25^+^ T cells under different stimulation conditions. Results of six independent experiments using CD4^+^CD25^+^ cells isolated from six different donors for (**D**) Ki-67 and (**E**) caspase-3, expressed as mean ± SD. p-values (* p<0.05, ** p<0.01 or ***p<0.001) are indicated with asterisks.

The cellular uptake of HSP70-FITC by Tregs was determined by immunofluorescence microscopy (Figure 1A–B). Unstimulated Tregs stained with DAPI (blue) were used as the negative control (Figure 1A). Microscopy results indicate that uptake of the protein and of the complex most probably occurred by HSP70 receptor-mediated endocytosis (Figure 1B).

The expression of the nuclear protein Ki-67 in T lymphocytes is a specific and quantitative indicator of proliferation *in vitro* [29]. Proliferating Tregs exhibit high levels of Ki-67, that is upregulated in the active cell cycle [30]–[32]. To determine Ki-67 levels on CD4^+^CD25^+^FoxP3^+^ Tregs in response to HSP70, IL-2 or the combination of both, we separated CD4^+^CD25^+^ from CD4^+^CD25^−^ cells and treated both cell subsets (Figure 1C and D). Levels of Ki-67 were significantly higher (up to 10-fold) in CD4^+^CD25^+^ T cells than in CD4^+^CD25^−^ T cells. In all donors tested, Ki-67 expression was significantly higher in CD4^+^CD25^+^ T cells treated with HSP70 alone or in combination with IL-2 compared to either untreated cells or cells treated with IL-2 alone. No significant change in Ki-67 expression occurred in the CD4^+^CD25^−^ T-cell subset (Figure 1C and D).

To further determine the effects of HSP70 and the combination of HSP70 plus IL-2 on apoptosis, we tested treated and untreated CD4^+^CD25^+^ and CD4^+^CD25^−^ cells for expression of intracellular caspase-3, a key protease activated during the early stages of apoptosis (Figure 1E) [32], [33]. There were only small differences between CD4^+^CD25^+^ and CD4^+^CD25^−^ cells (2.5%±0.9 to 5.9%±3.5) and the different donors in terms of caspase-3 expression, indicating that the investigated stimuli did not induce significant differences in apoptosis. In summary, the data show that exposure to HSP70 and in combination with IL-2 causes an increased proliferation of Tregs compared to non-Tregs.

### Cytokine secretion in IL-2-treated CD4^+^ T cells is increased by HSP70

**Figure 2:**
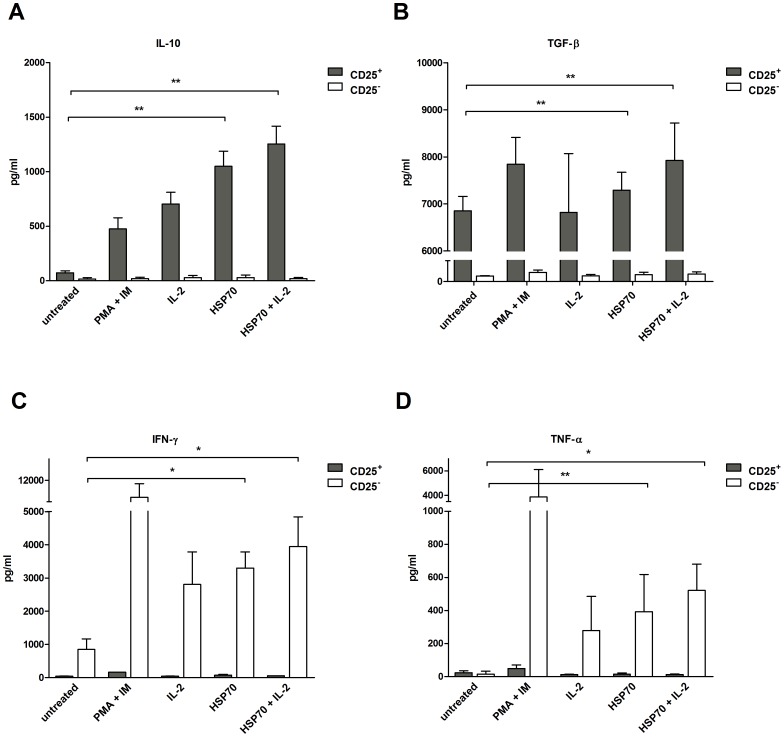
Augmentation of cytokine secretion by CD4^+^CD25^+^ and CD4^+^CD25^−^ T cells after HSP70 treatment. CD4^+^CD25^+^ and CD4^+^CD25^−^ T cells were incubated with IL-2 (200 U/ml), HSP70 (10 µg/ml) or both for 2 h, washed and transferred to 96-well plates coated with anti-CD3 antibodies (OKT3, 1 µg/ml) in serum-free medium. After 48 h of incubation, the supernatants were analyzed for (**A**) IL-10, (**B**) TGF-β, (**C**) IFN-γ and (**D**) TNF-α. Results of four independent experiments, expressed as mean ± SD. p-values (* p<0.05, ** p<0.01 or ***p<0.001) are indicated with asterisks.

To further investigate the effect of HSP70 on Treg activity, secretion levels of anti-inflammatory Treg suppressor cytokines (IL-10 and TGF-β) and proinflammatory target cell cytokines (IFN-γ and TNF-α) were measured in CD4^+^CD25^+^ and CD4^+^CD25^−^ T cells pretreated with either HSP70, IL-2 or a combination of these compounds (Figure 2). As a positive control T cells were stimulated with PMA/Iono [27].

IL-10 (Figure 2A) and TGF-β (Figure 2B) levels were significantly higher in HSP70-treated Treg cells as compared to untreated cells in all donors, but were highest in those cultures stimulated with HSP70 plus IL-2. As expected, CD4^+^CD25^−^ cells secreted almost no IL-10 and only small amounts of TGF-β, remained unresponsive to HSP70, and addition of IL-2 could not further enhance IL-10 and TGF-β secretion.

The observed changes in IFN-γ (Figure 2C) and TNF-α levels (Figure 2D) suggest that HSP70 strongly affects cytokine secretion on CD4^+^CD25^−^ cells. While there was little expression of IFN-γ and TNF-α in CD4^+^CD25^+^ cells, CD4^+^CD25^−^ cells secreted higher levels of these cytokines when treated with HSP70. Again, the addition of IL-2 enhanced these effects.

In conclusion, treatment with HSP70 or HSP70 plus IL-2 on CD4^+^CD25^+^ Tregs resulted in an increased secretion of the suppressor cytokines IL-10 and TGF-β. These results give the first evidence, that the suppressive capacity of Tregs is enhanced by HSP70.

### Proliferation of CD4^+^CD25^−^ target T cells is downregulated by HSP70-pretreated Tregs

**Figure 3:**
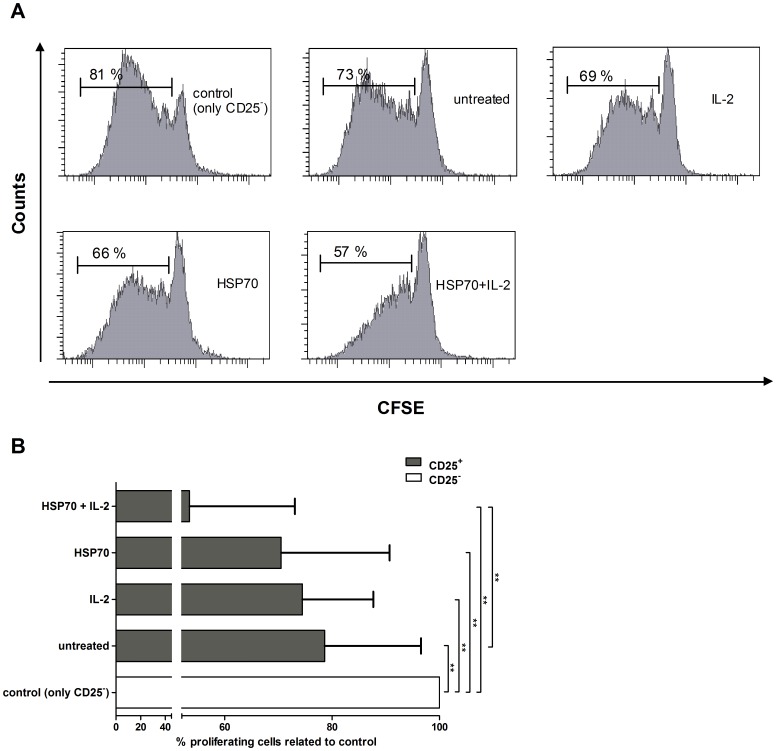
CD4^+^CD25^+^ treatment with HSP70 inhibits CD4^+^CD25^−^ target T-cell proliferation. CD4^+^CD25^+^ T cells were incubated with IL-2 (200 U/ml), HSP70 (10 µg/ml) or both for 2 h, washed and co-cultured with CFSE-labeled CD4^+^CD25^−^ target T cells (E∶T ratio 1∶10) on 96-well plates coated with anti-CD3 antibodies (OKT3, 1 µg/ml) in serum-free medium. For control CD4^+^CD25^−^ target T cells were culture in the absence of Tregs. After 5 days of incubation, cell proliferation was determined by FACS analysis. (**A**) One representative experiment of CD4^+^CD25^−^ T-cell proliferation under different CD4^+^CD25^+^ stimulation conditions. (**B**) Results of six independent experiments, expressed as mean ± SD. p-values (* p<0.05, ** p<0.01 or ***p<0.001) are indicated with asterisks. Proliferation levels of CD4^+^CD25^−^ target cells without Treg co-cultivation was defined as 100% (control, no suppression).

Immunosuppression via Tregs has been demonstrated to inhibit proliferation of CD4^+^ target T cells [34], [35]. To determine whether HSP70 affects the suppressive capacity of Tregs, isolated CFSE-labeled CD4^+^CD25^−^ target cells were co-cultured with untreated and treated (HSP70 and/or IL-2) effector CD4^+^CD25^+^ Tregs. Target cell proliferation was analyzed after 5 days of incubation (Figure 3) and target cell proliferation in a representative donor is shown in Figure 3A. For this representative donor, a CD4^+^CD25^−^ target cell proliferation of 81% was observed in the absence of effector Treg cells (control). Co-cultivation with IL-2-, HSP70- and HSP70 plus IL-2-pretreated Tregs led to a markedly reduced inhibition of target cell proliferation. The results in Figure 3B show that proliferation of CD4^+^CD25^−^ target cells was significantly inhibited in all donors tested. Proliferation levels of CD4^+^CD25^−^ target cells without Treg co-cultivation was defined as 100% (control, no suppression). By comparison, proliferation was downregulated to 78.6% (±17.9) after cultivation of target cells in the presence of untreated Tregs. When cultured in the presence of IL-2-or HSP70-pretreated Tregs, the proliferation capacity of the target cells decreased to 74.4% (±13.2) and 70.5% (±20.2), respectively. The strongest inhibition of target cell proliferation was induced by HSP70 plus IL-2-pretreated Tregs 53.4% (±19.6). Taken together the data show that HSP70- and HSP70 plus IL-2-primed Tregs have a significantly stronger inhibitory effect on target cell proliferation.

### Cytokine expression by CD4^+^CD25^−^ target cells in response to HSP70-pretreated Tregs

**Figure 4:**
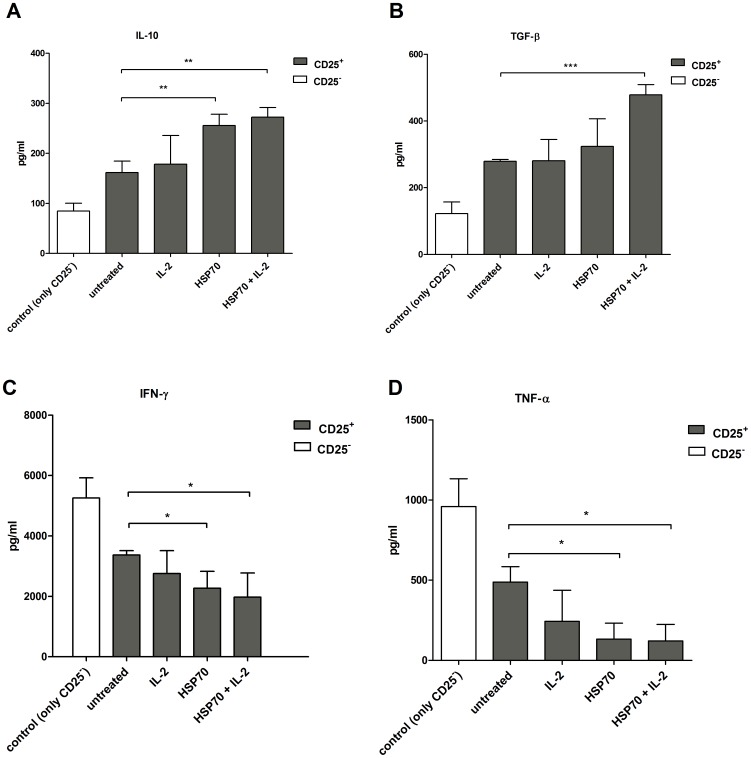
CD4^+^CD25^+^-induced inhibition of target T-cell proliferation is cytokine-dependent. CD4^+^CD25^+^ T cells were incubated with IL-2 (200 U/ml), HSP70 (10 µg/ml) or both for 2 h, washed and co-cultured with CFSE-labeled CD4^+^CD25^−^ target T cells (E∶T ratio 1∶10) on 96-well plates coated with anti-CD3 antibodies (OKT3, 1 µg/ml) in serum-free medium. For control CD4^+^CD25^−^ target T cells were culture in the absence of Tregs. After 5 days of incubation, the supernatants were analyzed for (**A**) IL-10, (**B**) TGF-β, (**C**) IFN-γ and (**D**) TNF-α. Results of six independent experiments, expressed as mean ± SD. p-values (* p<0.05, ** p<0.01 or ***p<0.001) are indicated with asterisks.

To further investigate the mechanisms of Treg-mediated downregulation of the CD4^+^CD25^−^ target cell activity, we analyzed the supernatants for expression of the suppressor cytokines (IL-10 and TGF-β) and inflammatory cytokines (IFN-γ and TNF-α) (Figure 4). Treg-mediated expression of IL-10 and TGF-β (Figure 4A and B) was low in CD4^+^CD25^−^ target cells cultured in the presence of untreated Tregs. Treg treatment with HSP70 alone and with HSP70 plus IL-2 caused a significant upregulation of the expression of these cytokines. Upregulation of IL-10 and TGF-β was also confirmed by mRNA expression levels (data not shown).

Similar to the upregulation of suppressor cytokines, downregulation of the target cell cytokines IFN-γ and TNF-α was observed (Figure 4C and D). CD4^+^CD25^−^ target cells cultured with untreated Tregs produced significantly higher levels of the proinflammatory cytokines than those cultured with HSP70 or HSP70 plus IL-2. The results are in concordance with those data obtained for the downregulation of target cell proliferation in response to HSP70-preactivated Tregs (Figure 3).

### Role of PI3K/AKT and MAPKs in HSP70-mediated activation of CD4^+^CD25^+^ Tregs

**Figure 5:**
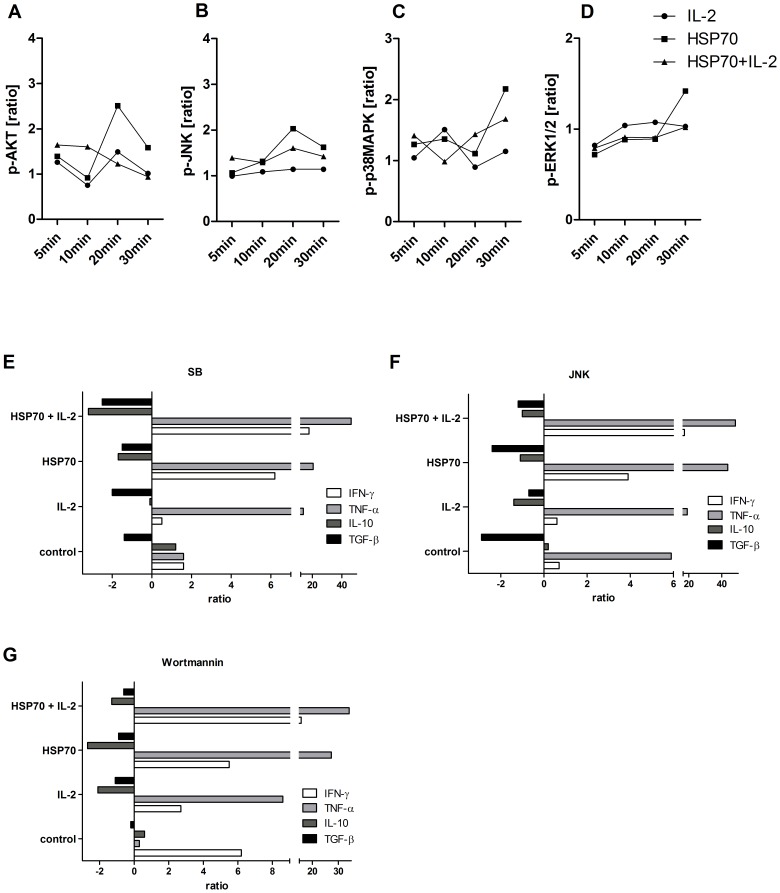
HSP70 mediates phosphorylation of AKT, p38, JNK and ERK1/2 in CD4^+^CD25^+^ T cells and suppressive capacity of these cells could be influenced by the respective inhibitors. CD4^+^CD25^+^ Treg cells were incubated alone, with IL-2 (200 U/ml), HSP70 (10 µg/ml) or both for 10 min washed and exposed to anti-CD3 antibodies (OKT3, 1 µg/ml) for 5, 10, 20 and 30 minutes. Phosphorylation for intracellular kinases (**A**) phospo-AKT [p-AKT Ser^473^], (**B**) phospo-JNK [p-JNK Thr^183^/Tyr^185^], (**C**) phospo-p38 MAPK [p-p38 MAPK Thr^180^/Tyr^182^] and (**D**) phospo-ERK1/2 [p-ERK1/2 Thr^202^/Tyr^204^/Thr^185^/Tyr^187^] was determined by the bead-based multiplex assay (Luminex xMAP technology). Untreated CD4^+^CD25^+^ T cells were adjusted to 1.00 by the use of the Bio-Plex Manager 6.0 software and used to calculate the ratios. Furthermore, CD4^+^CD25^+^ T cells were treated with 5 µM of the following intracellular signal transduction inhibitors: (**E**) Wortmannin, (**F**) JNK or (**G**) SB 203580 for 15 min before incubation alone, with IL-2 (200 U/ml), HSP70 (10 µg/ml) or both for 2 h. Cells were then co-cultured with CD4^+^CD25^−^ T cells (E∶T ratio 1∶5) on 96-well plates coated with anti-CD3 antibodies (OKT3, 1 µg/ml) in serum-free medium for 48 h. Supernatants were analyzed for IFN-γ, TNF-α, IL-10 and TGF-β. Results of four independent experiments, expressed as mean fold increases or decreases in comparison to results obtained for experiments without preactivation of Tregs and without inhibitor treatment (control).

PI3K/AKT plays a central role in T-cell survival and T-cell cytokine production [36]. Moreover, the MAPKs JNK, p38 and ERK1/2 are key regulators of cell proliferation and apoptosis [37]. Therefore, we investigated activation of these kinases upon stimulation with either IL-2, HSP70 alone or the combination of both compounds in CD4^+^CD25^+^ Tregs (n = 3). For a comparison we determined the activation of these kinases after stimulation via CD3 alone (Figure 5). No significant difference in phosphorylation of AKT (p-AKT, Ser^473^) by IL-2 alone was observed, whereas increased levels of p-AKT were determined upon HSP70 stimulation (Figure 5A). Stimulation with HSP70 plus IL-2 led to a maximum phosphorylation after 10 min which then decreased back to baseline levels.

The capacity of CD4^+^CD25^+^ Tregs to activate JNK (p-JNK, Thr^183^/Tyr^185^) was highest for HSP70-treatment after 20 min of stimulation (Figure 5B) and decreased in all experiments after 30 min. Co-cultivation of HSP70 plus IL-2 resulted in reduced p-JNK activation.

The level of phosphorylation of p38 (p-p38 MAPK, Thr^180^/Tyr^182^) fluctuates up and down up and down in all stimulation experiments (Figure 5C). Interestingly, HSP70- and HSP70 plus IL-2-stimulated Tregs activate p38 first upon 5 min of stimulation, whereas for IL-2-treated cells, an increase in p38 phosphorylation was observed after 10 min. A decline of p38 activation was observed after 20 min and increased again after 30 min of incubation for all three stimuli. The highest level of p-p38 was found after 30 min of stimulation with HSP70 alone.

Compared to activation by CD3 a downregulation of ERK1/2 phosphorylation (Thr^202^/Tyr^204^/Thr^185^/Tyr^187^) was observed within the first 5 min (Figure 5D) and could be approved after 60 min (Figure S3). Since we observed no activation of ERK1/2 for CD4^+^CD25^+^ Tregs after stimulation with HSP70 alone or in combination with IL-2 this pathway was not further considered in the inhibition experiments.

Taken together, these results indicate that stimulation of Tregs with HSP70 and HSP70 plus IL-2 resulted in the activation of PI3K/AKT, JNK and p38 MAP kinase pathways and did not influence the ERK1/2 signaling.

### Suppressive capacity of Tregs is influenced by specific inhibitors of signaling

To gain further insight into the immunomodulatory effects of HSP70 in Tregs we applied specific pharmacological inhibitors of AKT (Wortmannin, Figure 5E), JNK (JNK, Figure 5F), and p38 (SB 203580, Figure 5G). CD4^+^CD25^+^ Tregs (n = 4) were exposed to these inhibitors before TCR-stimulation via CD3 and treatment without any stimuli. These cells were co-cultured with CD4^+^CD25^−^ target cells (E∶T ratio 1∶5). The levels of IFN-γ and TNF-α as well as IL-10 and TGF-β were determined after 48 h of incubation. For control cells were cultured without inhibitors. Treatment of each of the three inhibitors led to blocked HSP70-enhanced downregulation of CD4^+^CD25^−^ target cells indicated as increasing levels of target cell cytokines IFN-γ and TNF-α. Direct inhibition of CD4^+^CD25^+^ Tregs could also be detected by decreasing levels of suppressor cytokines IL-10 and TGF-β after inhibition by the specific inhibitors. Therefore, the activation of PI3K/AKT, p38 and JNK signaling seems to be required for the augmented effects of CD4^+^CD25^+^ Tregs.

### Detection of target-independent granzyme B expression

**Figure 6:**
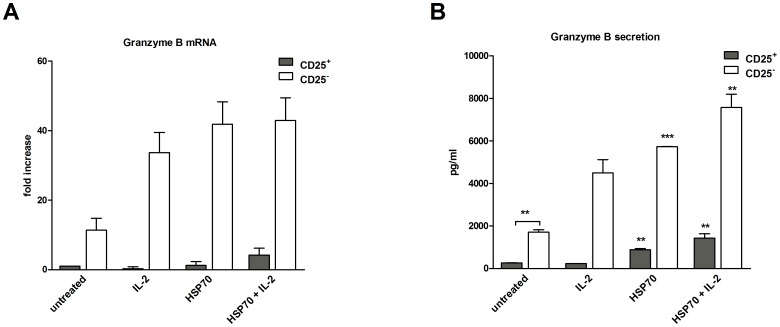
Secretion of cytotoxic effector molecule granzyme B of both CD4^+^CD25^+^ and CD4^+^CD25^−^ T-cell subsets is target-independent enhanced by HSP70. (**A**) Granzyme B mRNA levels in CD4^+^CD25^+^ T cells or independently stimulated CD4^+^CD25^−^ T cells were assessed by real-time PCR after 48 h of stimulation on anti-CD3 (OKT3, 1 µg/ml) coated plates in serum-free medium. Analysis was performed for comparison between the different CD4^+^CD25^+^ and CD4^+^CD25^−^ T-cell subsets. (**B**) Secretion of granzyme B was assessed by ELISA. Data from four independent experiments (mRNA) and two independent experiments (protein levels), expressed as mean ± SD. Comparison between groups was performed using t-tests. Statistically significant differences are indicated with asterisks (* p<0.05, ** p<0.01 or ***p<0.001).

HSP70 mediates cytotoxicity in CD4^+^ T cells, but the subtype of CD4^+^ T cells responsible for this cytotoxicity is unknown [24], [38]. To continue exposing CD4^+^ T-cell subsets to HSP70-mediated cytotoxic effects and to further demonstrate the impact of HSP70 on the function of Tregs, purified CD4^+^CD25^+^ Tregs and CD4^+^CD25^−^ T cells were incubated separately with either HSP70, IL-2 or both and analyzed for granzyme B mRNA and protein expression after 2 and 3 days, respectively of cultivation (Figure 6).

Treatment with HSP70 and/or IL-2 had only little effect on granzyme B mRNA levels in CD4^+^CD25^+^ Tregs but significantly higher effect in CD4^+^CD25^−^ T cells (Figure 6A). The highest increases occurred in CD4^+^CD25^−^ T cells treated with HSP70.

Granzyme B protein secretion levels in the supernatants were also measured by ELISA (Figure 6B). The highest levels of granzyme B were secreted by CD4^+^CD25^−^ T cells cultured in the presence of HSP70 and HSP70 plus IL-2. Significantly lower levels were observed in IL-2-treated or untreated CD4^+^CD25^−^ T cells. Granzyme B secretion by CD4^+^CD25^+^ Tregs was much lower but still significantly higher in the presence of HSP70 or HSP70 plus IL-2 than in IL-2-treated or untreated cells.

These results demonstrate that HSP70 can augment a target-independent increase in the secretion of the cytotoxic effector molecule in all of the studied T-cell populations, showing the most pronounced effect in the CD4^+^ non-Treg target cell population.

## Discussion

In the current study the role of HSP70 on the function and modulation of Tregs and their potential to suppress target effector T cells *in vitro* is investigated. It is demonstrated that activation by extracellular HSP70 enhances the ability of Tregs to secrete suppressor cytokines, suppress secretion of target cell cytokines and inhibit the proliferation of CD4^+^CD25^−^ target cells. Our results also showed that PI3K/AKT and the MAPKs p38 and JNK appeared to be involved in the regulatory pathways activated by HSP70 upon TCR-mediated stimulation via CD3. Furthermore, it is shown that HSP70 is able to enhance the cytotoxic capacity of both CD4^+^ T-cell subsets by an induction of granzyme B. CD4^+^CD25^−^ T cells were clearly identified as the CD4^+^ T-cell subset which is mainly responsible for the increased HSP70-dependent granzyme B expression.

### HSP70-mediated functional activation of Tregs

The ability of Tregs to effectively downregulate the function of different T-cell subsets has previously been demonstrated *in vitro* and *in vivo* [34], [39]. It is, however, still an open question how Tregs can be treated to boost or repress their suppressive capability (at least *in vitro*) to modulate immune responses. Therefore, we examined whether the exposure to HSP70 might be involved in Tregs activity. Individual stimulation of both CD4^+^CD25^+^ Tregs and depleted CD4^+^CD25^−^ target T cells with HSP70 prior to TCR activation led to a function of both T-cell subsets as demonstrated by an increased expression of IL-10 and TGF-ß (CD4^+^CD25^+^ T cells) and of IFN-γ and TNF-α (CD4^+^CD25^−^ T cells), respectively (Figure 2). The results of our study are in accordance with those of others showing the immunogenic potential of HSP70 in T cells [5], [40], [41]. However, all previous studies described immunogenic abilities of HSP70 and other HSPs only on effector T cells such as CD4^+^, CD8^+^ T cells or NK cells. The HSP effects on Tregs remained largely unclear – except in the case of HSP60. HSP60 was shown to interact with Tregs via TLR2 by enhancing their ability to express IL-10 and TGF-ß and to downregulate target T-cell proliferation [23]. Similar to these results, by using anti-TLR2 and anti-TLR4 antibodies, we found that mainly TLR2 but not TLR4 is able to mediate the enhancing effects of human HSP70 on the regulatory activity of human Tregs (Figure S2). Like HSP70, HSP60 belongs to the group of “high molecular weight” heat shock proteins. While HSP60 is mainly located in mitochondria, HSP70 has been found within the cytosol and inside the nucleus.

We clearly demonstrated the effects of HSP70 on Tregs, underscoring its immunologic potential and extending the findings identifying HSP70 as an important modulator of the innate immune response. Here, it is shown that HSP70-pretreated Tregs are able to inhibit proliferation in CD4^+^CD25^−^ target T cells. CD25^+^FoxP3^+^ Tregs account just 5–10% of the total CD4^+^ T-cell subset in humans and animals. Hence, we performed our proliferation assays at an E∶T ratio of 1∶10 to simulate the proportion of Tregs to target cells under more physiological conditions. Without HSP70 pretreatment, Tregs showed low but still significant downregulation of target cell proliferation, whereas pretreatment with HSP70 was able to broadly augment the observed downregulation (Figure 3).

In 2001, Multhoff *et al.* described a N-terminal-extended 14-mer HSP70-derived peptide TKDNNLLGRFELSG (TKD, aa 450–463) which was able to stimulate the cytolytic and proliferative activity of natural killer (NK) cells at concentrations equivalent to full-length HSP70 protein [42]. Further studies are required to determine which domains may be involved in the interaction of HP70 and regulatory T cells. It is likely that those HSP-derived peptides can be used in a clinical setting to increase the efficiency and functionality of the cells before adoptive transfer.

As the clinical success in treating transplant rejection today has been achieved primarily through therapeutic donation of immunosuppressive drugs more selective therapies that focus only on the pathologic immune responses without affecting protective function of the immune system remain a major intention in clinical transplantation. At this point, HSP70 may play a key role in understanding and achieving this intention. HSP70 has already been shown to inhibit acute rejection in a Treg-dependent mechanism [43]. Furthermore, first clinical trials promise HSP70 to be effective in reduction of acute allograft rejection [44].

The underlying mechanisms which might be involved in inhibition of the target cell activities by Tregs are controversially discussed. Some investigators reported that Treg function requires cell-cell contacts, while others have reported that the suppressive cytokines IL-10 and TGF-β were mainly responsible for their effects [35], [45]–[47]. Again, it has been demonstrated that both contact-dependent and cytokine-dependent effects are involved in these mechanisms [23]. We found evidence of Treg effects such as increased cytokine levels of IL-10 and TGF-β secreted by Tregs and decreased IFN-γ and TNF-α cytokine levels produced by downregulated target cells (Figure 3). Although we focused on the cytokine secretion mechanisms, we cannot rule out that contact-dependent effects of Tregs may mediate suppressing the proliferation of target T cells.

### HSP70- induced phosphorylation of PI3K/AKT and the MAPKs JNK and p38 but downregulated ERK phosphorylation

HSPs (e.g., HSP60, HSP70 or gp96) have been shown to interact with T cells mainly via TLR2 and TLR4 [23], [48], [49]. While cell surface receptors and co-stimulatory molecules are well-known and described [50]–[54], the signal transduction cascades in Tregs in response to stress stimuli are largely unkown. Because of limited cell numbers, isolation of pure populations, lack of suitable Treg staining markers as well as the lack of pure endotoxin-free HSP preparations these studies are often hampered. Here, we demonstrate that PI3K/AKT, p38 and JNK MAPKs are involved in HSP70-dependend activation of Tregs, while the MAPK ERK1/2 had a significantly lower impact on Tregs. By determining intracellular signaling events in Tregs after exposure of the cells to HSP70, IL-2 and a combination of both stimuli we found differences in the phosphorylation status of proteins (Figure 4). It was reported, that human Tregs might have a defect in phosphorylation of AKT at serine 473 following TCR stimulation resulting in a decreased activity of downstream effectors [55]. The authors hypothesized that a reduced activity of AKT might be responsible to maintain the suppressive capacity of CD4^+^CD25^+^ Treg cells [55]. Similar to earlier findings we observed a reduced activation of PI3K/AKT, MAPKs p38 and JNK after stimulation with HSP70 alone or in combination. Interestingly in all experiments co-cultivation with IL-2 showed inhibition in phosphorylation activity for all analyzed pathways.

In addition, we found no increase in ERK1/2 activation in CD4^+^CD25^+^ Tregs compared to CD3-mediated TCR activation, which have been also shown previously for *in vitro* expanded Tregs derived from cord blood [56]. Our data showed for the first time, that under the influence of HSP70 AKT, p38 as well as JNK showed increased kinase activity. Co-cultivation with IL-2 resulted in a decreased phosphorylation of the kinases but induced an increase in the suppressive capacity of the cells, which was shown by inhibition of the respective pathways, indicating a complex interplay between the different pathways under stress conditions.

### Cytolytic effects of HSP70-treated Tregs

Granzyme B is a well-known cytolytic molecule found in specific granules of CD8^+^ and CD4^+^ CTLs as well as in NK cells; it also lyses tumor cells and virus-infected cells [57]. We recently demonstrated that exposure to extracellular HSP70 can lead to a more cytotoxic phenotype of CD4^+^ T cells [24]. In contrast, the functional properties of CD8^+^ CTLs did not seem to be affected by the protein. Interestingly, Gondek et al. identified granzyme B as a key component of Treg-mediated cytolytic effects [58]. In the present study we examined which CD4^+^ T-cell subset is mainly responsible for the increasing granzyme B expression by HSP70-stimulated CD4^+^ T cells. Beyond a doubt, we demonstrated that CD4^+^CD25^−^ cells are the CD4^+^ population responsible for augmentation of granzyme B levels in a similar amount to CD8^+^ CTLs [24]. However, effects of HSP70 on the cytotoxic properties of CD4^+^CD25^+^ Tregs could also be demonstrated here.

## Conclusion

Tregs have been the focus of basic science and clinical research in autoimmune disease and allograft transplantation and have been shown to play a major role in balancing immune reactions and allograft tolerance [59]. Specifically the immunosuppressive influence on target T cells has been implicated to ameliorated autoimmune mediated tissue destruction and allograft rejection. One promising concept is thus, to use Tregs as cell therapeutics with the aim to replace immunosuppressive therapy in autoimmune disease and allograft transplantation. Understanding the mechanisms of Treg-mediated immunomodulation as well as identifying ways of expansion and optimizing their function is therefore paramount for their future use in the treatment of patients. As it is known, that the physiological amount of CD25^+^FoxP3^+^ Tregs accounts just 5–10% of the total CD4^+^ T-cell subset in humans we would assume that HSP70 used in a clinical scenario may most likely also lead to an unintentional activation of effector autoimmune T cells. Because this could be significant in a potential physiologic or pathologic clinical scenario the *ex vivo* modulation of and expansion of Tregs from graft recipients with HSP70 and IL-2 could be a first step to induce improved long-term outcomes and represent a new therapeutic strategy in the cell and organ transplantation setting and in inflammatory diseases of autoimmune origin.

## Supporting Information

Figure S1 **Dose-dependent activation of CD3^+^ and CD4^+^ T cells by HSP70.** Purified CD3^+^ and CD4^+^ T cells were treated with increasing concentrations of HSP70 for 2 h, washed and transferred to 48-well plates coated with anti-CD3 antibodies (OKT; 1 µg/ml) in serum-free medium. IFN-γ, TNF-α and IL-10 levels in the supernatants were determined after 20 h of incubation. The effect of extracellular HSP70 was investigated in CD3^+^ and CD4^+^ T-cell subsets to determine the optimal working concentration of HSP70. As treatment of T cells prior to activation appears to be significantly more effective, freshly isolated purified CD3^+^ and CD4^+^ T cells (purity >98%) were treated with different concentrations of HSP70 (0.001–50 µg/ml) prior to treatment with anti-CD3 antibodies. Secretion of the pro-inflammatory cytokines IFN-γ (Figure 1A) and TNF-α (Figure 1B) as well as the anti-inflammatory cytokine IL-10 was then analyzed (Figure 1C). HSP70 upregulates IFN-γ, TNF-α and IL-10 secretion in both CD3^+^ and CD4^+^ T cells in a dose-dependent manner. The highest cytokine levels were observed using HSP70 at concentrations above 10 µg/ml. In order to evaluate if the obtained results are specific for HSP70 we used recombinant endotoxin-free beta2 microglobulin (B2M) in the same concentrations as control. Using this protein we could not detect any effect on the cells (data not shown). Significant sections (0.1–10 µg/ml) of the results of four independent experiments, expressed as mean ± SD. p-values (* p<0.05, ** p<0.01 or ***p<0.001) are indicated with asterisks.
(TIF)

Figure S2 **Effects of HSP70 on CD4^+^CD25^+^ T_regs_** **are dependent on TLR2 signaling.** Isolated CD4^+^CD25^+^ T cells were pretreated with monoclonal anti-TLR2 or anti-TLR4 antibodies (eBioscience, San Diego, USA) (20 µg/ml, 60 minutes). Then, the cells were incubated alone, with HSP70 (10 µg/ml) or a combination of HSP70 plus IL-2 (200 U/ml) for 2 h, co-cultured with CFSE-labeled CD4^+^CD25^−^ target T cells (E∶T ratio 1∶10), washed and transferred to 96-well plates coated with anti-CD3 antibodies (OKT; 1 µg/ml) in serum-free medium. For control CD4^+^CD25^−^ target T cells were culture in the absence of Tregs. After 5 days of incubation, cell proliferation was determined by FACS analysis (Figure 2A). IFN-γ (Figure 2B) and granzyme B (Figure 2C) levels in the supernatants were determined after 48 h of incubation. To gain further insight in Treg signaling we pretreated CD4^+^CD25^+^ regulatory T cells with neutralizing anti-TLR2 or anti-TLR4 antibodies before treatment with HSP70 or HSP70 plus IL-2 to determine the influence of TLR mediated signaling in Tregs. We found that anti-TLR2 but hardly anti-TLR4 was able to block the enhancing effects of HSP70 as shown by reduced Treg suppression of T effector cell proliferation (Figure 2A). We could further show that anti-TLR2 but not anti-TLR4 significantly blocked the Treg mediated suppression of IFN-γ secretion by T effector cells (Figure 2B). To further extend our findings we also determined cytotoxic effects of those cells by measuring granzyme B levels. Here we also detected anti-TLR2 effects on Tregs as demonstrated by fold increases of granzyme B levels in anti-TLR2 blocked samples compared to those only with untreated Tregs (HSP70: 3.16±2.68 and HSP70+IL-2: 3.21±2.82 fold increase) (Figure 2C). Results of four independent experiments, expressed as mean ± SD. p-values (* p<0.05) are indicated with asterisks.
(TIF)

Figure S3 **Role of ERK1/2 signaling in CD4^+^CD25^+^ T_regs_.** In order to expand our findings on the ERK1/2 pathway in Tregs we used 1×10^6^ purified CD4^+^CD25^+^ T cells (>92% FoxP3^+^), stimulated them for 10 min with IL-2 (200 U/ml), HSP70 (10 µg/ml) or a mixture of both. The cells were then washed and replated in the same concentration on anti-CD3 mAb- (OKT3, 1 µg/ml) precoated 24-well plates for 3, 5, 10, 20, 30 and 60 min (37°C, 5%CO_2_). Total cell lysates derived from the cell culture samples were prepared according to the manufacturer's instructions (BioRad, Hercules, USA). Phosphorylation of the two target proteins (ERK1/2 and p38) in CD4^+^CD25^+^ T-cell subsets was detected using the bead-based BioPlex phosphoprotein detection assay (BioRad). Samples were analysed on a Luminex-200 instrument using Bio-Plex Manager 6.0 software (BioRad). For Western Blot Analysis cells were lysed and analyzed for phospho (p)-ERK1/2, p-p38MAPK, total-ERK1/2 and total-p38MAPK, while GAPDH was used as a control. Briefly, membrane was blocked in 5% milk Tris-buffered saline with Tween (TBST, 10 mMTris pH 8.0, 150 mMNaCl, and 0.05% Tween-20) at room temperature for 1 h. Primary antibody (against phospho-ERK1/2, phospho-p38MAPK, ERK1/2 and p38MAPK (Santa Cruz, California, USA) was diluted at 1∶1000 in 5% milk/TBST and incubated with the membrane overnight at 4°C. The secondary antibody (horseradish peroxidise conjugated goat anti-rabbit) (Santa Cruz, California, USA) was added at 1∶2000 in 5% milk/TBST at room temperature for 1 h. Membrane was stained with TMB Blotting substrate solution (Kem-En-Tec, Taastrup, Denmark ). The modified diagram of p-ERK1/2 (Figure S3A) clearly demonstrates a reduced activation level. We demonstrated a negative or borderline-low activation status of ERK1/2 compared to p-p38MAPK. Moreover, our additional data set generated by Western Blot (Figure S3A) and flow cytometry (Figure S3B) confirm the previous observation obtained by Luminex assay (Figure 5D). Studies which were focused on specific signaling have demonstrated the maximal activation of p-ERK1/2 after 5 min and 15 min [1], [2]. For these time points, we observed no p-ERK1/2 activation in CD4^+^CD25^+^ Tregs by using all three detection assays (Luminex, Western Blot, flow cytometry). No specific spots were detected by Western Blot after 15 min of stimulation, while flow cytometry analysis and Luminex also resulted in a reduced activation level of p-ERK1/2 after 3–60 min of stimulation. The highest p-ERK1/2 level was detected after 30 min HSP70 stimulation by flow cytometry and Luminex, whereas that activation level was significant lower than those of p-p38. Studies had shown that the TCR-mediated signaling pathway in Tregs included defective phosphorylation of ITMAs within the CD3ζ chain which results in a reduction of ERK phosphorylation, JNK activation and a failure to phosphorylate AKT at Ser^473^ [3]. As excepted we also observed reduced activation levels of p-AKT, p-JNK and p-p38, whereas they were significant higher than those of p-ERK1/2. These data clearly indicate that p-ERK1/2 is not being activated after 3–60 min of stimulation, while p-AKT, p-JNK and p-p38 have a reduced activation level, indicating defects in the activation/phosphorylation of component from the TCR-mediated signaling pathway in human Tregs.
(TIF)
