# Supplementary figures and images for: HSP70 Enhances Immunosuppressive Function of CD4+CD25+FoxP3+ T Regulatory Cells and Cytotoxicity in CD4+CD25− T Cells
Source: PLoS One. 2012 Dec 26;7(12):e51747. doi: 10.1371/journal.pone.0051747 (PMC3530531; doi:10.1371/journal.pone.0051747)

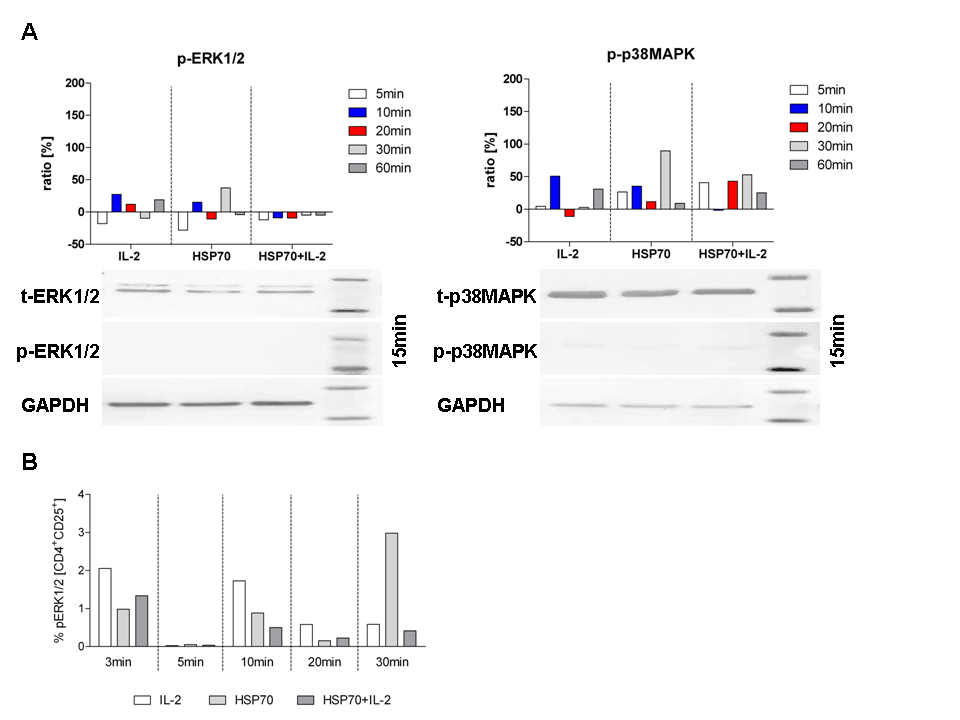

Supplement: Figure S3 — Role of ERK1/2 signaling in CD4+CD25+ Tregs. In order to expand our findings on the ERK1/2 pathway in Tregs we used 1×10^6^ purified CD4+CD25+ T cells (>92% FoxP3+), stimulated them for 10 min with IL-2 (200 U/ml), HSP70 (10 µg/ml) or a mixture of both. The cells were then washed and replated in the same concentration on anti-CD3 mAb- (OKT3, 1 µg/ml) precoated 24-well plates for 3, 5, 10, 20, 30 and 60 min (37°C, 5%CO2). Total cell lysates derived from the cell culture samples were prepared according to the manufacturer's instructions (BioRad, Hercules, USA). Phosphorylation of the two target proteins (ERK1/2 and p38) in CD4+CD25+ T-cell subsets was detected using the bead-based BioPlex phosphoprotein detection assay (BioRad). Samples were analysed on a Luminex-200 instrument using Bio-Plex Manager 6.0 software (BioRad). For Western Blot Analysis cells were lysed and analyzed for phospho (p)-ERK1/2, p-p38MAPK, total-ERK1/2 and total-p38MAPK, while GAPDH was used as a control. Briefly, membrane was blocked in 5% milk Tris-buffered saline with Tween (TBST, 10 mMTris pH 8.0, 150 mMNaCl, and 0.05% Tween-20) at room temperature for 1 h. Primary antibody (against phospho-ERK1/2, phospho-p38MAPK, ERK1/2 and p38MAPK (Santa Cruz, California, USA) was diluted at 1∶1000 in 5% milk/TBST and incubated with the membrane overnight at 4°C. The secondary antibody (horseradish peroxidise conjugated goat anti-rabbit) (Santa Cruz, California, USA) was added at 1∶2000 in 5% milk/TBST at room temperature for 1 h. Membrane was stained with TMB Blotting substrate solution (Kem-En-Tec, Taastrup, Denmark ). The modified diagram of p-ERK1/2 (Figure S3A) clearly demonstrates a reduced activation level. We demonstrated a negative or borderline-low activation status of ERK1/2 compared to p-p38MAPK. Moreover, our additional data set generated by Western Blot (Figure S3A) and flow cytometry (Figure S3B) confirm the previous observation obtained by Luminex assay (Figure 5D). Studies which were focuse [file pone.0051747.s003.tif]
